# Silver Nanoparticle Surface Enabled Self-Assembly of Organic Dye Molecules

**DOI:** 10.3390/ma12162592

**Published:** 2019-08-14

**Authors:** Hua Deng, Hongtao Yu

**Affiliations:** Department of Chemistry, School of Computer, Mathematical and Natural Sciences, Morgan State University, Baltimore, MD 21251, USA

**Keywords:** silver nanoparticle, self-assembly, fluorescence quenching, self-assembly shielding effect, fluorescent organic molecules

## Abstract

Fluorescence titration of methylene blue, rhodamine B and rhodamine 6G (R6G) by silver nanoparticle (AgNP) all resulted in an initial steep quenching curve followed with a sharp turn and a much flatter quenching curve. At the turn, there are about 200,000 dye molecules per a single AgNP, signifying self-assembly of approximately 36-layers of dye molecules on the surface of the AgNP to form a micelle-like structure. These fluorescence-quenching curves fit to a mathematical model with an exponential term due to molecular self-assembly on AgNP surface, or we termed it “self-assembly shielding effect”, and a Stern-Volmer term (nanoparticle surface enhanced quenching). Such a “super-quenching” by AgNP can only be attributed to “pre-concentration” of the dye molecules on the nanoparticle surface that yields the formation of micelle-like self-assembly, resulting in great fluorescence quenching. Overall, the fluorescence quenching titration reveals three different types of interactions of dye molecules on AgNP surface: 1) self-assembly (methylene blue, rhodamine B and R6G), 2) absorption/tight interaction (tryptamine and fluorescein), and 3) loose interaction (eosin Y). We attribute the formation of micelle-like self-assembly of these three dye molecules on AgNP to their positive charge, possession of nitrogen atoms, and with relatively large and flat aromatic moieties.

## 1. Introduction

Metal nanoparticles (NP) have shown great potential for use in catalysis [1], sensing [2], imaging [3,4] and antimicrobials [5,6]. Over 400 tons of silver nanoparticle (AgNP) have been produced annually and 30% of them are used in medical applications due to their antibacterial properties [7]. Most of these nanoparticle-containing products will be released to the environment and ultimately conduct unique interactions with biomolecules, such as the formation of protein and environmental corona [8,9]. These interactions are a result of the surface interactions between AgNP and ambient species or through surface-enabled molecular interactions. Hence, it is of interest to understand AgNP surface-enabled molecular interactions and reactions, which are also fundamental to the understanding of the toxicity of AgNP [10]. 

There have been reports on the study of the interaction between AgNP and biologically active small molecules such as dopamine [11], melamine [12], and pyronin Y [13], and organic molecules containing thiol groups [14]. In general, AgNPs have the highest binding affinity to thiol containing molecules [14,15]. Log K of –SR to Ag(I) is 12, compared to 2.1 and 3.4 for –OR and –NH groups, respectively [16]. Munro et al. [17] studied the surface of citrate coated AgNP using SERS, they suggested that citrate’s two carboxylate groups bind the surface Ag atoms of the AgNP while its tertiary hydroxyl group forms intermolecular hydrogen bonds, and with the third carboxylate acting as a reaction site. Petty et al. [18] confirmed that cysteine moieties are the main sites for AgNP attachment in DNAs. Zhao et al [19] reported that rhodamine 6G (R6G) molecules can form dimers on AgNP surface which is related to localized surface plasmon resonance. These findings open some insights into the molecular interaction on the surface of AgNP. 

Due to the lack of effective instruments or methods, it is difficult to obtain direct information on the interaction between AgNP and small molecules. To estimate the number of small molecules bound to AgNP, one has to go through a complex process: AgNP-organic molecule complexes are often separated from free organic molecules in solution using centrifugation, followed by mass spectroscopy or UV-vis spectrometry to examine the amount of organic molecules bound to AgNP surface. The number of molecules bound to each AgNP can be estimated by comparing the concentration differences of these molecules before and after mixing with AgNP [6,20,21]. X-ray structure was used to study the structure of the crystalized complex of a gold nanocluster (102 Au atoms) with p-mercaptobenzoic acid (p-MBA), which showed that 44 p-MBA molecules bind to 23 surface Au atoms [22]. The resolution was 1.1 Å. However, there have been no such studies for AgNP [23,24,25]. Currently, only surface plasmon resonance and SERS [12,17] have been used to investigate AgNP interaction with small molecules, while theoretical calculations based on density functional theory or first principles quantum theory [12,26] complement the experimental studies.

Previously, we reported a micelle-like structure formation between R6G molecules and AgNP utilizing fluorescence titration [27]. Each “micelle” consists of approximately 210,000 R6G molecules assembling on a single 24 nm AgNP surface under certain conditions. The size growth from citrate-coated AgNP (24 nm) to micelles (170 nm) and micelle agglomerates (370 nm) was confirmed by dynamic light scattering (DLS). In order to understand whether this kind of self-assembly is applicable in other dye molecules and the factors govern such formation, we choose five more dye molecules, rhodamine B, methylene blue, fluorescein, eosin Y and tryptamine with distinct structural features, to study their interaction with AgNP through fluorescence titration, a method also used to investigate interactions between proteins and metal NP [28,29]. 

## 2. Methods

Silver nitrate, sodium borohydride, sodium citrate, R6G, rhodamine B, methylene blue, fluorescein, eosin Y, and tryptamine were purchased from Sigma Aldrich (St. Louis, MO). Nitric acid (trace metal grade, 67–70%) was from Fisher Scientific (Houston, TX).

AgNPs were prepared following a previous reported method [6,27,30] using sodium borohydride to reduce silver nitrate, followed by citrate mediated reduction and growth [31,32]. Briefly, silver nitrate (17 mg) and sodium borohydride (15 mg) were each dissolved in 90 mL ice-chilled nanopure water. The two solutions were mixed slowly and a yellowish color appeared. The mixture was left for 5 min under stirring followed by addition of 10 mL of sodium citrate (70 mg). The resultant solution was heated and kept under reflux at 90–95 °C for 30 min. After cooling to room temperature, the solution was centrifuged at 5000 rpm for 1 h and the pellet was collected and suspended into 20 mL water. Centrifugation was repeated twice to remove extra reactants. Finally, a further centrifugation at 2000 rpm for 30 min to remove the largest NPs was carried out and the supernatant was collected and kept in the refrigerator as AgNP stock solutions for further experiments.

The AgNP was characterized by UV-vis spectroscopy (UV-2600 spectrophotometer, Shimadzu, Kyoto, Japan) and Transmission Electron Microscopy (TEM, JOEL 2100, Tokyo, Japan). AgNPs have a characteristic extinction peak at 398 nm. The TEM images show that citrate coated AgNPs have spherical morphology of an average diameter of 24 ± 7 nm.

To determine the concentration of AgNP, its solution was added with nitric acid to completely oxidize all Ag atoms to Ag^+^ ions. The resultant Ag^+^ solution was diluted with nanopure water until the estimated Ag^+^ concentration ranged from 20 to 100 ppb. It was then analyzed by ICP-MS (Varian 820-MS, Palo Alto, USA) to determine the total concentration of Ag^+^ ion and thus the concentration of the AgNP in terms of Ag atoms.

The number of Ag atoms in each AgNP was estimated by the following equation [33,34],

(1) $$N=\frac{\pi }{6}\frac{{\rho d}^{3}}{M}N_{A}=\frac{{\pi N}_{A}d^{3}}{6V_{m}}=30.67d^{3}$$

where ρ is the density of Ag (10.49 g/cm^3^), M is the atomic weight of Ag (107.87 g/mol), V_m_ is molar volume (10.5 mL mol^−1^), d is the diameter of AgNP in nm. It yields 420,000 Ag atoms per one 24 nm AgNP. Therefore, the concentrations in terms of Ag atoms that are obtained from ICP-MS can be converted to concentrations in terms of AgNP. E.g., 100 μM in term of Ag atoms equals to 2.4 × 10^−4^ μM or 240 pM in term of AgNP. Concentrations used below are in terms of AgNP.

Two concentrations of dye molecules, 1 μM and 10 mM, were used for fluorescence titrations. Fluorescence parameter of different dyes and instrumental setup are shown in Table 1. AgNP (240 pM or 2400 pM) were titrated into 2,000 μL of dye solutions of 1 μM and 10 μM, respectively, with the following volumes of 0, 2.5, 2.5, 2.5, 2.5, 5.0, 5.0, 5.0, 5.0, 10.0, 10.0, 10.0, 10.0, 10.0, 10.0, 20.0, 20.0, 20.0, 20.0, 20.0, 40.0, 40.0, 40.0, and 40.0 μL. Emission spectra were recorded and fluorescence intensity values at the peak were collected and analyzed, using Fluoromax-4 spectrofluorometer (Horiba Scientific, Kyoto, Japan). The excitation spectra were also monitored upon addition of AgNP, following the same volume. 

When AgNP solutions are gradually added into the 1 µM or 10 µM dye solutions, it causes dilution to the original dye solution and thus lower the fluorescence intensity. The dilution factor, d_f_, was used to correct the fluorescence intensity before the plot of fluorescence quenching curve, following Equations (2) and (3).

(2) $$d_{f}=\frac{2000\;+\;\sum_{0}^{n}V_{i}}{2000}$$

F_actual_ = F_observed_ × d_f_ (3)

V_i_ is the volume of AgNP solution added each time in the unit of µL, d_f_ is the dilution factor. F_actual_ and F_observed_ are fluorescence intensities used for analysis and collected from flurometer, respectively. F_actual_ is the true fluorescence and used as F in the further analysis. Concentration of Ag^+^ ions released from AgNP is very low (around 100 nM level) [6] and their quenching to R6G fluorescence is less than 1% [35]. This is confirmed by using AgNO_3_ solution of equivalent concentration as a control quencher and it shows no effect on fluorescence of dye molecules. Therefore, the role of Ag^+^ ions on fluorescence quenching is ignored in the current study.

DLS was employed to monitor the change of dynamic size and zeta-potential during titrations, using a Zetasizer Nano-ZS (Malvern, UK).

## 3. Results and Discussion

### 3.1. Effect of AgNPs on the Fluorescence of Dye Molecules

**Figure 1:**
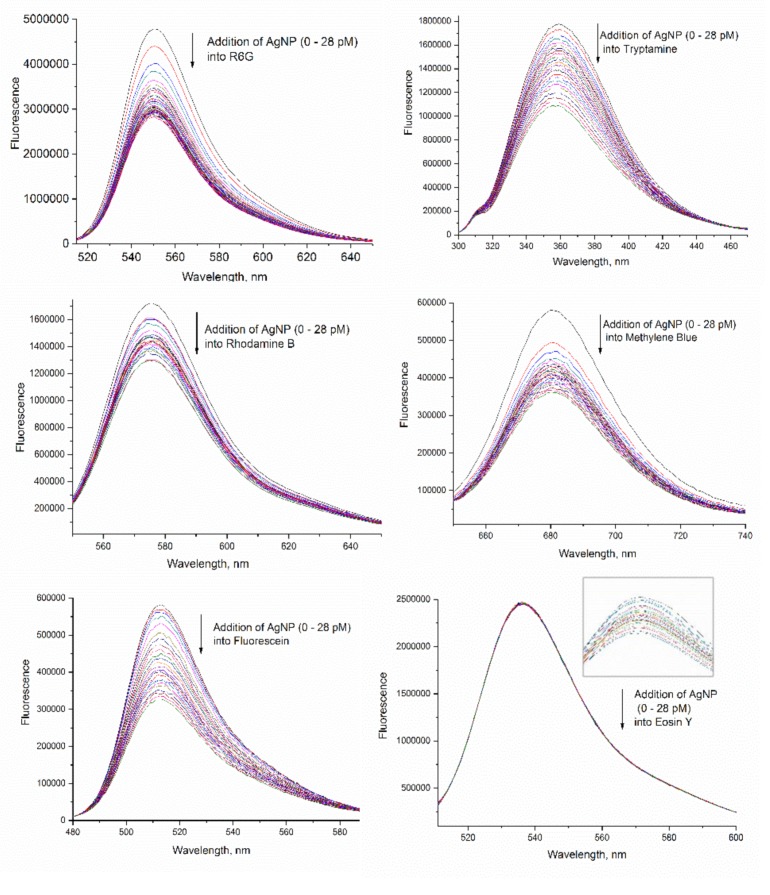
Fluorescence quenching of 1 μM dye solutions upon addition of various amounts of 240 pM AgNP. The insert for eosin Y shows the enlarged detail of fluorescence peaks, where comparatively weak quenching is observed.

Fluorescence spectra of 1 μM dye solutions upon addition of various amounts of the 240 pM AgNP solutions are shown in Figure 1. As AgNP solutions are gradually added to the solution of the dyes, the fluorescence intensity of the dyes decreases, an indication of quenching, although the rates of fluorescence decrease are different for different dyes. The emission spectra for all the dyes remain the same during titration except tryptamine, whose emission maximum shifts to a shorter wavelength from 359 to 356 nm. The titration into the 10 μM dye solutions also show similar fluorescence quenching patterns (data not shown) except that emission peak of tryptamine has a larger shift from 359 nm to 345 nm.

**Figure 2:**
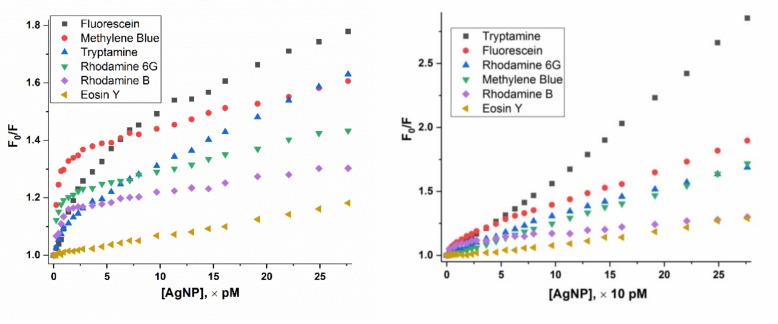
Plot of fluorescence intensity ratio (F_0_/F) versus AgNP concentration at dye concentration of 1 μM (left) and 10 μM (right). Average value from five measurements are shown and error bars are removed for a better comparison.

Effect of the addition of AgNP on fluorescence of various dye molecules in solution is analyzed by the Stern-Volmer plot, or plotting the fluorescence intensity ratio (F_0_/F) versus AgNP concentration. The AgNP concentration on the x-axis of titration for the 10 µM dye solutions is also 10 times higher than that of the 1 µM dye solution (Figure 2). The higher is the F_0_/F ratio, the more fluorescence is quenched. 

In the case of 1 µM dye solution, the F_0_/F of methylene blue and rhodamine B increases sharply at the initial stage followed by a distinct turn for a slower increase at the AgNP concentration around 1–2 pM, the same way as that of R6G. As we proposed for R6G, where a micelle-like self-assembly is formed with 210,000 R6G molecules assembling on the surface of one AgNP [27], we believe methylene blue and rhodamine B molecules also assemble on the surface of AgNP under these conditions to form such micelles. As for eosin Y, the fluorescence quenching is at the very minimum. The plot for tryptamine and fluorescein, whose fluorescence ratios increase slowly, but steadily. Ultimately, they reach the highest quenching among all dyes tested.

When the concentration of the dye molecules is 10 μM, the fluorescence of all dye molecules in solution is also quenched, without the sharp turn on the plots for R6G, methylene blue and rhodamine B observed when the dye solution is 1 μM. This indicates that the micelle-like self-assembly of these dye molecules on AgNP surface does not occur when the dye solution is at 10 μM. Fluorescein and tryptamine exhibit a much smoother quenching curve, which is also distinct with those of 1 μM dye solution. Eosin Y shows similar pattern since its quenching is weak.

Both absorption and excitation spectra were obtained for the titration. Due to low sensitivity of the photospectrometer, the absorption has to be done at 20 µM (or above) of the dye molecules (Figure S1 of Supplementary Materials shows spectra of 50 µM dyes). When 2400 pM of AgNP solution were titrated into 50 μM dye solution, R6G and methylene blue cause AgNP aggregation, while tryptamine, rhodamine B, fluorescein and eosin Y do not (Figure S1). In term of absorption intensity, tryptamine, rhodamine B, fluorescein and eosin Y do not exhibit obvious decrease. The excitation spectra showed no shifts for all dye molecules (Figure S2 of Supplementary Materials). 

### 3.2. Different Behaviors of the Dyes on AgNP Surface

Methylene blue and rhodamine B as 1 μM solutions show a similar quenching behavior with that of R6G, with a sharp quenching at initial additions of AgNP until a sharp turn, followed by a much slower quenching. This demonstrates that methylene blue and rhodamine B have the same capability to self-assemble as R6G on the AgNP surface to form stable micelle-like structures. 

Fluorescence of fluorescein or tryptamine is gradually quenched with a smaller slope compared with R6G, rhodamine B and methylene blue, but lacks the “turn”. These results indicate that fluorescein and tryptamine may gather around AgNP core in an absorption/tight interaction mode in a dynamic equilibrium but no stable micelle-like structure is formed since no obvious turn is observed (Figure 2).

Eosin Y maintains a small but stable quenching slope during the whole titration.

These three types of molecular behaviors are further confirmed by the change of dynamic size and zeta-potential (Table 2). Rhodamine 6G and Rhodamine B have steady but dramatic change of both diameter and zeta-potential. Fluorescein and tryptamine have relative small size change with a difference around 34 nm and 36 nm, respectively. Size of AgNP in the presence of eosin Y is slightly increased due to “pre-concentration” that is further discussed in Section 3.2.3. Based on these interaction patterns, two mathematical models are used to fit the plot of F_0_/F versus [AgNP] using Origin 2017 SR2.

#### 3.2.1. Stern-Volmer Fluorescence Quenching Model for eosin Y

**Figure 3:**
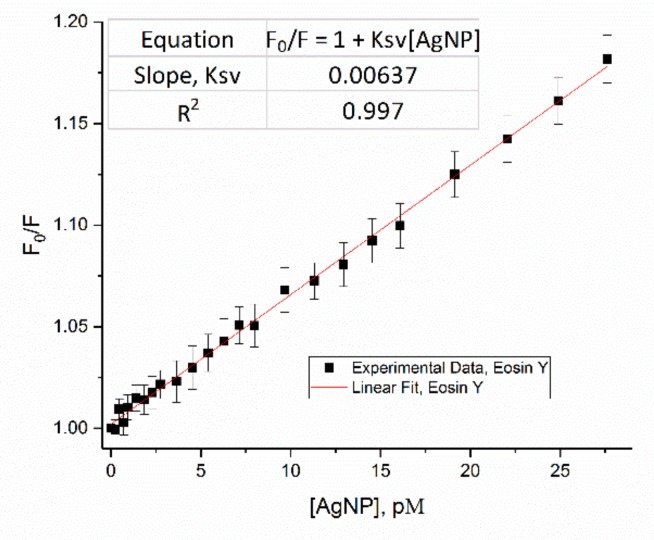
Fit of F_0_/F verse (AgNP) plot of eosin Y.

For eosin Y, its fluorescence-quenching plot, F_0_/F verse [AgNP], fits very well to the Stern-Volmer equation (Figure 3), with K_sv_ of 6.4 nM^−1^ of [AgNP], or 15,000 M^−1^ of [Ag] based on the estimate that one AgNP of 24 nm has about 420,000 Ag atoms.

Considering the Stern-Volmer equation,

(4) $$\frac{F_{0}}{F}=1+k_{q}\tau_{0}\left[\mathrm{AgNP}\right]$$

the quenching rate constant k_q_ is comprised of two terms, the fraction of collisions that result in quenching f_Q_, and the diffusion controlled bimolecular rate constant k_0_.

(5) $$k_{q}=f_{Q}k_{o}$$

(6) $$k_{0}=\frac{4{\pi N}_{0}}{1000\frac{{\mathrm{cm}}^{3}}{L}}\left(r_{f}+r_{q}\right)\left(D_{f}+D_{q}\right)$$

where N_0_ is Avogadro’s number, r_f_ and r_q_ are the radii of the fluorophore and quencher, and D_f_ and D_q_ are the diffusion coefficients of the fluorophore and quencher, respectively. Based on the Stokes-Einstein equation

(7) $$D=\frac{K_{B}\times T}{6\pi \eta r}$$

where K_B_ is Boltzmann constant, T is absolute temperature, η is the dynamic viscosity and r is the particle size. Diffusion coefficient (D) is mainly dependent on the particle size. Under current experimental conditions, eosin Y has a much smaller size than AgNP whose diffusion coefficient can be ignored when compared to that of eosin Y. Thus, Equation (4) can be written as:

(8) $$\frac{F_{0}}{F}=1+f_{Q}\tau_{0}D_{f}\frac{4{\pi N}_{0}}{1000\frac{{\mathrm{cm}}^{3}}{L}}\left(r_{f}+r_{q}\right)\left(\mathrm{AgNP}\right)$$

Eosin Y has lifetime τ_0_ of 2.50 ns [36] and small organic dye molecules normally have diffusion coefficients D_f_ around 420 µm^2^ s^−1^ [37]. Given approximate radius of eosin Y and AgNP as 0.8 nm and 12.0 nm, equation [6] yields an f_Q_ of 6.17 × 10^−20^, demonstrating an extremely low percentage of collisions that cause fluorescence quenching. 

In contrast, Eosin Y has an extremely high K_sv_ of 6.4 nM^−1^ of [AgNP], or 15,000 M^−1^ of [Ag],compared with the normal small molecule quenching, like the fluorescence quenching of tryptamine by acrylamide in an aqueous solution, which is around 33 M^−1^ [38]. Thus, the terms of traditional dynamic/collisional quenching and static quenching are not proper to describe fluorescence quenching by nanoparticles (See Figure S3 for lifetime measurement). Instead, we employed “nanoparticle surface enabled fluorescence quenching” to describe fluorescence quenching of eosin Y by AgNP. 

#### 3.2.2. Mathematics Model for the Molecular Self-Assembly on AgNP Surface

**Figure 4:**
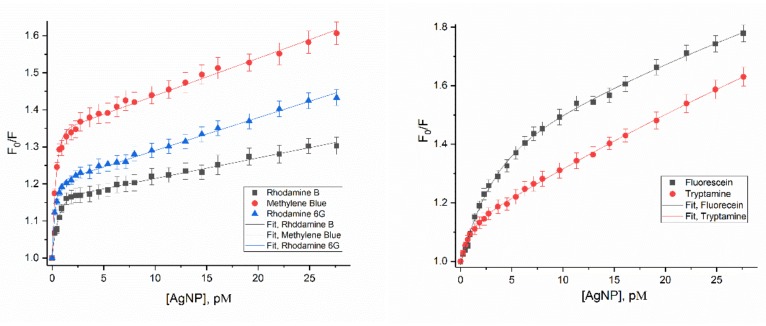
Fit of F_0_/F verse [AgNP] plot of 1 µM R6G, rhodamine B, methylene blue, fluorescein and tryptamine.

Plots of F_0_/F of rhodamine B, R6G, and methylene blue versus [AgNP] all fit very well to a function composed of an exponential term (self-assembly shielding effect) plus a Stern-Volmer term (nanoparticle surface enhanced fluorescence quenching), shown in Figure 4 and Equation (9).

(9) $$\frac{F_{0}}{F}=A\left(1-e^{-\frac{\left(\mathrm{AgNP}\right)}{B}}\right)+\left(1+K_{SV}\left(\mathrm{AgNP}\right)\right)$$

The resulting constants *A, B,* and K_sv_ are listed in Table 2 from the excellent fits. 

The initial exponential term represents the rapid quenching through self-assembly shielding effect, where the internal dye molecules of the micelles cannot absorb light and thus not get to the excited state to fluoresce, due to the self-assembly of the dye molecules on the surface of AgNP. Constant *A* corresponds to the AgNP concentration at which a stable micelle-like structure is in an equilibrium with the free dye molecules in solution. The higher is the *A* value, the lower is the concentration required for the corresponding dye to reach equilibrium between stable micelle-like structures and those free in solution. Thus, three dyes that form stable micelle-like structures follow a formation sequence from fastest to lowest: methylene blue > R6G > rhodamine B (*A* values of 0.33, 0.20 and 0.16, respectively). 

Constant *B* represents the relative binding affinity between AgNP and dye molecules. Smaller *B* values indicate stronger interactions, and larger numbers of dye molecules gathering around each AgNP at equilibrium, and ultimately self-assembly shielding effect as well as fluorescence quenching percentages. The three dyes follow an interaction affinity sequence of rhodamine B < methylene blue < R6G (*B* values of 0.59, 0.38 and 0.33, respectively).

The fluorescence quenching of R6G, rhodamine B and methylene blue by AgNP have an obvious turning point where stable “micelle”-like structures are formed. Equation (5) is employed to estimate the number of dye molecules that assemble on the surface of each AgNP, assuming that all the fluorescence quenching is caused by interactions between dye molecules and the AgNP.

(10) $$\frac{\mathrm{Quenched}\;\mathrm{Dye}}{\mathrm{AgNP}}=\frac{2000\;\mu L\;\times \;1\;\mu M\;\times {\;N}_{0}\frac{I}{I_{0}}}{240\;\mathrm{pM}\;\times {\;N}_{0}\sum_{0}^{n}V_{i}}=\frac{10^{8}\;\times \;\frac{I}{I_{0}}}{12\;\times \;\sum_{0}^{n}V_{i}}$$

V_i_ is the value of each titration volume, with unit of µL. At the turning point, this ratio demonstrates the number of molecules in a single stable micelle-like structure, in other words, the number of dye molecules that a stable “micelle” can “hold” in a dynamic equilibrium. The estimated numbers are listed in Table 3, with 180,000, 200,000, and 220,000 for rhodamine B, methylene blue, and R6G, respectively, per AgNP. This number indicates that at dynamic equilibrium, the majority of the dye molecules in solution are assembled on AgNP. The remaining small portion is close to the “minimum” concentration of free dye molecules to maintain balance in the solution.

The Stern-Volmer term K_sv_, representing the collisional quenching, has values for rhodamine B, R6G, and methylene blue of 5.5, 8.9 and 10 nM^−1^ of [AgNP], respectively, or 13,000, 21,000, and 24,000 M^−1^ of Ag atoms.

**Figure 5:**
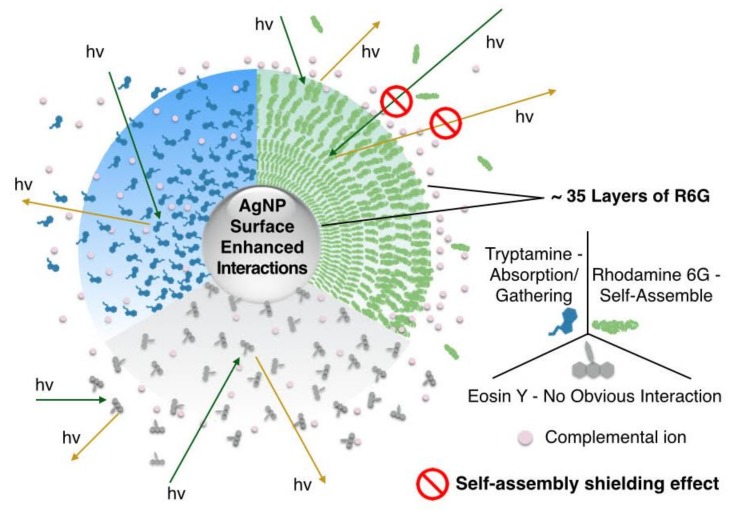
Schematic drawing of different types of interaction and behavior of various dye molecules, and “self-assembly shielding effect” caused by self-assemble of Rhodamine 6G molecules near AgNP surface.

The fluorescence quenching graphs for tryptamine and fluorescein also fit to Equation (9), but due to the lack of the sharp turn, there is no indication of a definitive “micelle” formed. Therefore, there is no estimate made for the number of molecules per AgNP. The fitting parameters *A, B,* and K_sv_ are on the same order of magnitude with those for methylene blue, rhodamine B and R6G, but the parameters *B* and K_sv_ values are obviously larger (Table 3). This demonstrates an absorption/tight interaction mode on AgNP surface and “pseudo-micelles” could be formed with these dyes (Figure 5).

#### 3.2.3. “Super” Stern-Volmer Fluorescence Quenching Constant

One interesting observation is that the Stern-Volmer quenching constant (nanoparticle surface enhanced fluorescence quenching) for all the six dye molecules by AgNP are generally three orders of magnitude higher than the normal small molecule quenching, like the fluorescence quenching of tryptamine by acrylamide in an aqueous solution, which is around 33 M^−1^ [38], making AgNP a super-quencher [39]. One explanation is that the dye molecules, except those forming the micelles, are in close proximity of AgNP, or “pre-concentrated” on the AgNP surface. During this process, fluorescence quenching is a complicated result caused by a combined self-assembly shielding effect and traditional static and even dynamic quenching. This again indicates that these dye molecules, charged or neutral, are in the proximity of the AgNP.

### 3.3. “Self-Assembly Shielding Effect” Caused Fluorescence Quenching

It is well known that aggregation, including self-assembly, causes fluorescence quenching of dye molecules in aqueous solution (it is sometimes termed “aggregation caused fluorescence quenching, ACQ”), owing to electron or energy transfer [40,41,42]. It occurs under relatively high concentrations for aggregation, at 10 µM or higher [42,43]. In our case, the self-assembly of dye molecules on AgNP surface indicates that traditional electron or energy transfer (among dye molecules) causes fluorescence quenching, even under 1 µM for both the dye and AgNP. However, it cannot explain the “sharp turn” of the plot for R6G, methylene blue and rhodamine B. 

Taking R6G as an example, 210,000 R6G molecules assemble on a single AgNP (24 nm) with an estimated 35 layers (See SI 3, Figure S3, Table S1 and S2 for estimation details) [27]. As shown in Figure 5, the inner layers of R6G would be shielded from absorbing light and fluoresce [44], which we term “self-assembly shielding effect” to describe the unique fluorescence quenching caused by self-assembly of dye molecules on AgNP surface. Considering that 35 layers of dye molecules, R6G, methylene blue or rhodamine B are tightly surrounding the AgNP core, the excitation light could penetrate approximately the outmost one to several layers (due to gaps among dye molecules). It cannot travel through many layers of self-assembled dye molecules to reach those of inner layers. Therefore, inner layers of dye molecules are shielded from excitation by light and thus would not generate any fluorescence. This is a unique fluorescence quenching mechanism caused by AgNP enabled self-assembly behavior, with a characteristic “sharp turn” on the plot of fluorescence intensity ratio (F_0_/F) versus AgNP concentration (Figure 2).

For dye molecules that have loose or no obvious interaction with AgNPs, like eosin Y in Figure 5, the pathway of light is not completely blocked so that its fluorescence quenching is mainly caused by electron transfer, FRET and/or NSET. Some organic molecules show a fluorescence quenching efficiency between R6G and eosin Y, indicating they have a gathering around AgNP but it is not as dense/thick, nor as well-organized as that of R6G. Self-assembly shielding effect may also be involved but it is not that obvious. 

### 3.4. Factors Affecting Molecular Interactions on AgNP Interface

#### 3.4.1. Concentration of both Dyes and Nanoparticles

**Figure 6:**
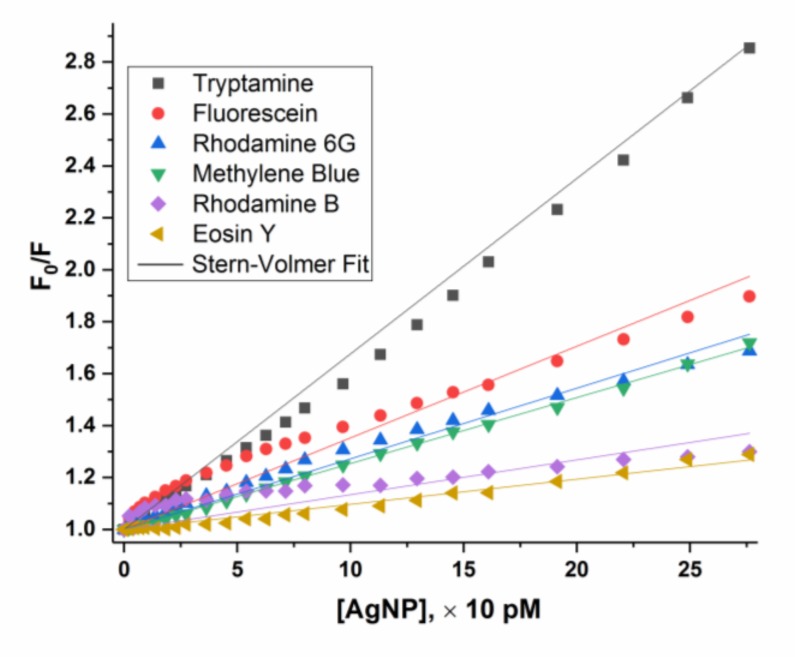
Fit of F_0_/F verse [AgNP] plot of dye molecules in the case of 10 µM. Error bars are removed for a better comparison.

It is obvious that the self-assembly of dye molecules on AgNP surface is concentration dependent; and it only occurs at low concentrations for both AgNP and dyes. For the dye solutions of 10 µM, all quenching plots fit to a pseudo Stern-Volmer plot (Figure 6) with K_sv_ values of 10–68 nM^−1^ [AgNP] (Table 4), higher than those constants for dyes in the 1 µM solutions. Such large Stern-Volmer quenching constants still indicate super-quenching. At the [AgNP] /[dye] ratio of 30, the quenching strength from the most to the least quenched dyes are: fluorescein > tryptamine ≈ methylene blue > rhodamine 6G > rhodamine B > eison Y for 1 µM dye solutions; and tryptamine > fluorescein > methylene blue ≈ rhodamine 6G > rhodamine B ≈ eison Y for 10 μM dye solutions. It is obvious that concentration plays an important role on fluorescence quenching by AgNP. 

#### 3.4.2. Intrinsic Molecular Structure

**Figure 7:**
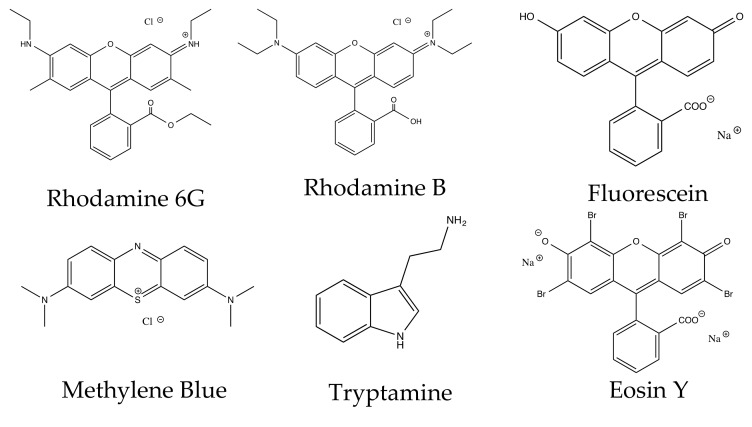
Structures of tested dye molecules.

The structures of all tested dyes are shown in Figure 7 and their properties are listed in Table 5 with information on charge, functional groups and paired ions. Self-assembly is determined from interactions between AgNP and dye molecules at 1 µM as shown in Figure 2. 

#### 3.4.3. Charge

All dye molecules forming the micelle-like self-assemblies on AgNP have a positive charge, indicating positive charge promotes the self-assembly of the dyes on AgNP. Surface of citrate coated AgNP are negatively charged, thus AgNP favors binding to positively charged molecules. It is rather unusual that these positively charged aromatic dye molecules self-assemble on the AgNP surface to form large micelles.

#### 3.4.4. Functional Groups

In addition to the positive charge, rhodamine B, R6G and methylene blue all have some nitrogen atoms in the molecule. We think that the nitrogen group also helps to facilitate the micelle formation. The other feature of these three molecules are the relatively large and flat aromatic moieties, which might be necessary for the micelle formation as we discussed in our previous publication with R6G (30). However, it is difficult to pinpoint the exact roles the functional groups play in the self-assembly, as well as the “pre-concentration” on the AgNP surface. These results are in agreement with previous reports that AgNP have preferable binding sites in terms of specific functional groups and other protein components or secondary structure [12,15,45,46]. 

## 4. Conclusions

Furthermore, the self-assembly can only occur at low concentrations (at 1 µM or lower) for both dye and AgNP, while at a higher dye concentration (10 µM) yields pseudo Stern-Volmer quenching curves. Although the quenching of fluorescein and tryptamine by AgNP also fits to the same two-term model, the quenching curve lacks the signature “sharp turn” for the formation of the “micelle-like” self-assembly. The quenching of eosin Y follows the Stern-Volmer quenching model very well without the exponential term. The Stern-Volmer quenching constants (K_sv_) for these dyes are in the range of 1.5–10 nM^−1^ of [AgNP], or 13,000–43,000 M^−1^ of [Ag], three orders of magnitude higher than the K_sv_ value of the collisional quenching of tryptamine fluorescence by acrylamide (33 M^−1^).

Such a “super-quenching” by AgNP can only be attributed to “pre-concentration” of the dye molecules on the nanoparticle surface. The “pre-concentration” of methylene blue/R6G/rhodamine B near the surface of AgNP further yields the formation of micelle-like self-assembly, resulting in even greater fluorescence quenching during the initial stage of the titration. Detailed mechanisms of self-assembly remain to be explored but the common features of all these dye molecules are the positive charge, the aromatic moiety, and the polar groups. The charge of the dye molecules seems to be the dominant factor. Negatively charged or neutral molecules are not likely to self-assemble on the AgNP surface. The fluorescence quenching for Methylene Blue, Rhodamine B, and Rhodamine 6 G all fit to a mathematical model with an exponential term (self-assembly) and a Stern-Volmer term (nanoparticle surface enhanced quenching). 

These findings and models provide fundamental information of nanoparticle surface enabled interaction and molecular behavior, which could further facilitate the understanding of protein and environmental corona formation, and benefit engineering nanoparticle-based nanomaterials.

## Figures and Tables

**Table 1:** Parameter and instrumental setup for fluorescence titrations.

Dye	Excitation Wavelength, nm	Slit Width, nm, 1 µM	Slit Width, nm, 10 µM	Emission Peak, nm
Rhodamine 6G	500	1	1	554
Rhodamine B	540	2	1	580
Methylene Blue	640	5	4	688
Fluorescein	470	1	1	513
Eosin Y	505	2	1	539
Tryptamine	280	2	2	356

**Table 2:** Dynamic diameter and zeta-potential of AgNP coated by citrate and in presence of six dye molecules.

	AgNP with citrate	Rhodamine 6G	Rhodamine B	Methylene Blue *	Fluorescein	Eosin Y	Tryptamine
Size, nm	33.4 ± 1.6	318.5 ± 34.4	197.6 ± 62.1	/	67.1 ± 12.4	42.8 ± 3.9	69.2 ± 13.0
Potential, mV	−47.3 ± 1.2	21.5 ± 2.0	17.6 ± 1.8	/	−16.9 ± 2.2	−31.8 ± 1.6	8.9 ± 3.7

Notes: Data at the titration point of 2 pM AgNP is shown. At this point, the corresponding dye reach equilibrium between stable micelle-like structures or complexes and free molecules in solution. * DLS results of methylene blue are not available due to interference between laser source wavelength of DLS (633 nm) and absorption of methylene blue (peaked at 612 nm and 664 nm).

**Table 3:** Mathematical fitting results for all dye molecules.

	$\frac{F_{0}}{F}=A(1-$ $e^{-\frac{\left[AgNP\right]}{B}})+\left(1+K_{sv}\left[AgNP\right]\right)$						
Dye		*A*	*B*	K_sv_, nM^−1^ (AgNP)	K_sv_, M^−1^ (Ag)	R^2^	Dyes/AgNP
Rhodamine B		0.16	0.59	5.5	13,000	0.992	180,000
Rhodamine 6G		0.20	0.33	8.9	21,000	0.996	220,000
Methylene Blue		0.33	0.38	10	24,000	0.995	200,000
Fluorescein		0.37	3.37	1.5	36,000	0.998	/
Tryptamine		0.12	1.27	1.8	43,000	0.999	/
Eosin Y		/	/	6.4	15,000	0.997	/
Tryptamine*		/	/	/	33*	/	/

* The K_sv_ for tryptamine quenching by acrylamide is 33 M^−1^ [38].

**Table 4:** Stern-Volmer fit results (K_sv_ in nM^−1^ of [AgNP]) with the concentration of the dyes at 10 µM.

	Tryptamine	Fluorescein	R6G	Methylene Blue	Rhodamine B	Eosin Y
K_sv_	68	35	27	25	13	10

**Table 5:** Properties of dye molecules and their fluorescence quenching behavior.

Dye	Charge	Functional Groups	Pair Ions	Self-Assembly
Rhodamine 6G	1+	-NH-, -N=, -O-, -COO-	Cl^-^	Yes
Rhodamine B	1+	-N-, -N=, -O-, -COO-	Cl^-^	Yes
Methylene Blue	1+	-N-, -N=, -S-	Cl^-^	Yes
Fluorescein	1-	-OH, -COO-, -O-, =O	Na^+^	Weak
Eosin Y	2-	-COO-, –Br, -O-, =O	Na^+^	No
Tryptamine	0	-NH-, -NH_2_	NA	Weak

## Supplementary Materials

The following are available online at https://www.mdpi.com/1996-1944/12/16/2592/s1. Supporting information includes UV-Vis spectra of 50 µM dye solutions titrated with AgNP and excitation spectra of 1 µM dye titrated with AgNP. Figure S1: UV-vis spectra of titration of 1.18 nM AgNP (equivalent to 500 µM in terms of Ag atoms) into 50 μM dye solutions of rhodamine B (RB), methylene blue (MB), rhodamine 6G (R6G), tryptamine (TA), fluorescein (Flus), eosin Y (EY). The same dilution factor (titration of nanopure water) is used as control. The number represent the total volume of AgNP solution added, Figure S2: Excitation spectra of 1 µM dye solutions titrated with 240 pM AgNP. The numbers represent the volume (µL) of every single addition, Figure S3: A cuboidal R6G molecule with a dimension of 1.7 nm × 1.1 nm × 0.4 nm. Extended dashes of 0.3 nm indicate the minimal distance between neighboring R6G molecules in the assembly. This yields an actual dimension of 2.0 nm × 1.4 nm × 0.7 nm within the assembly, Figure S4: Fluorescence decay of 1 μM R6G alone (above) and that in the presence of citrate-coated AgNP (below, R6G to AgNP ratio of 8.48×105), Table S1: Theoretical estimation of the number of layers and total numbers of R6G molecules on AgNP surface in a micelle-like structure, Table S2: Number of R6G molecules whose fluorescence is quenched by a single AgNP, and their ratio, possible number of layers, as well as the dynamic diameter of the micelle-like structure.
