# Supplementary material for: Silver Nanoparticle Surface Enabled Self-Assembly of Organic Dye Molecules
Source: Materials (Basel). 2019 Aug 14;12(16):2592. doi: 10.3390/ma12162592 (PMC6720720; doi:10.3390/ma12162592)
Supplement: Supplementary file 1 [file materials-12-02592-s001.pdf]

materials

MDPI

Supplemental Materials

Silver Nanoparticle Surface Enabled Self-assembly of Organic Dye Molecules

Hua Deng and Hongtao Yu *

Department of Chemistry, School of Computer, Mathematical and Natural Sciences, Morgan State University, Baltimore, Maryland, 21251

* Correspondence: hongtao.yu@morgan.edu; Tel.: 443-885-4515

Received: 30 July 2019; Accepted: 13 August 2019; Published: 21 August 2019

SI 1: UV-Vis spectra 50 µM dye solutions titrated with AgNP

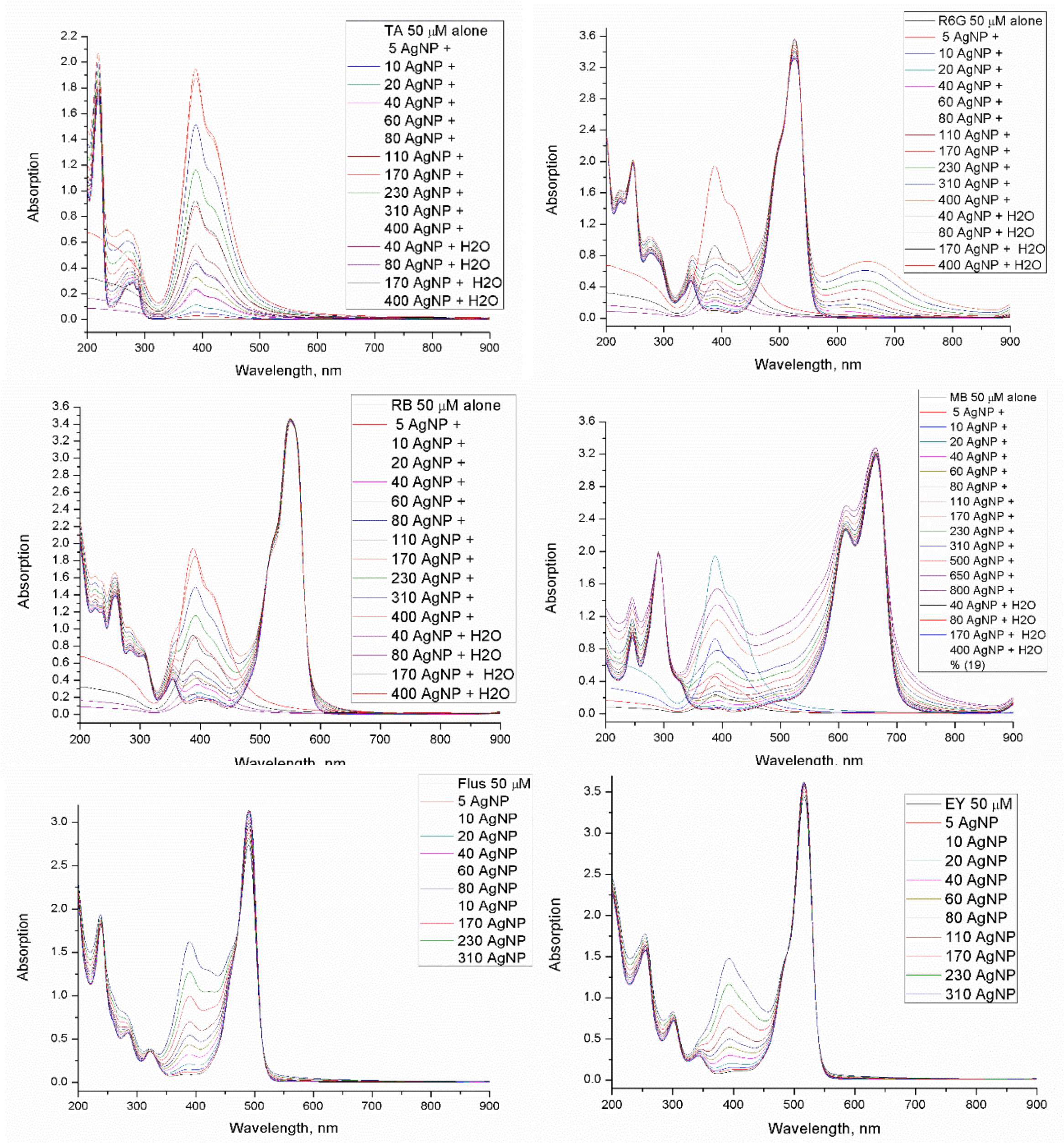

Figure S1. UV-vis spectra of titration of 1.18 nM AgNP (equivalent to 500 µM in terms of Ag atoms) into 50 µM dye solutions of rhodamine B (RB), methylene blue (MB), rhodamine 6G (R6G), tryptamine

(TA), fluorescein (Flus), eosin Y (EY). The same dilution factor (titration of nanopure water) is used as control. The number represents the total volume of AgNP solution added.

SI 2. Excitation spectra of 1 μM dye solutions titrated with AgNP

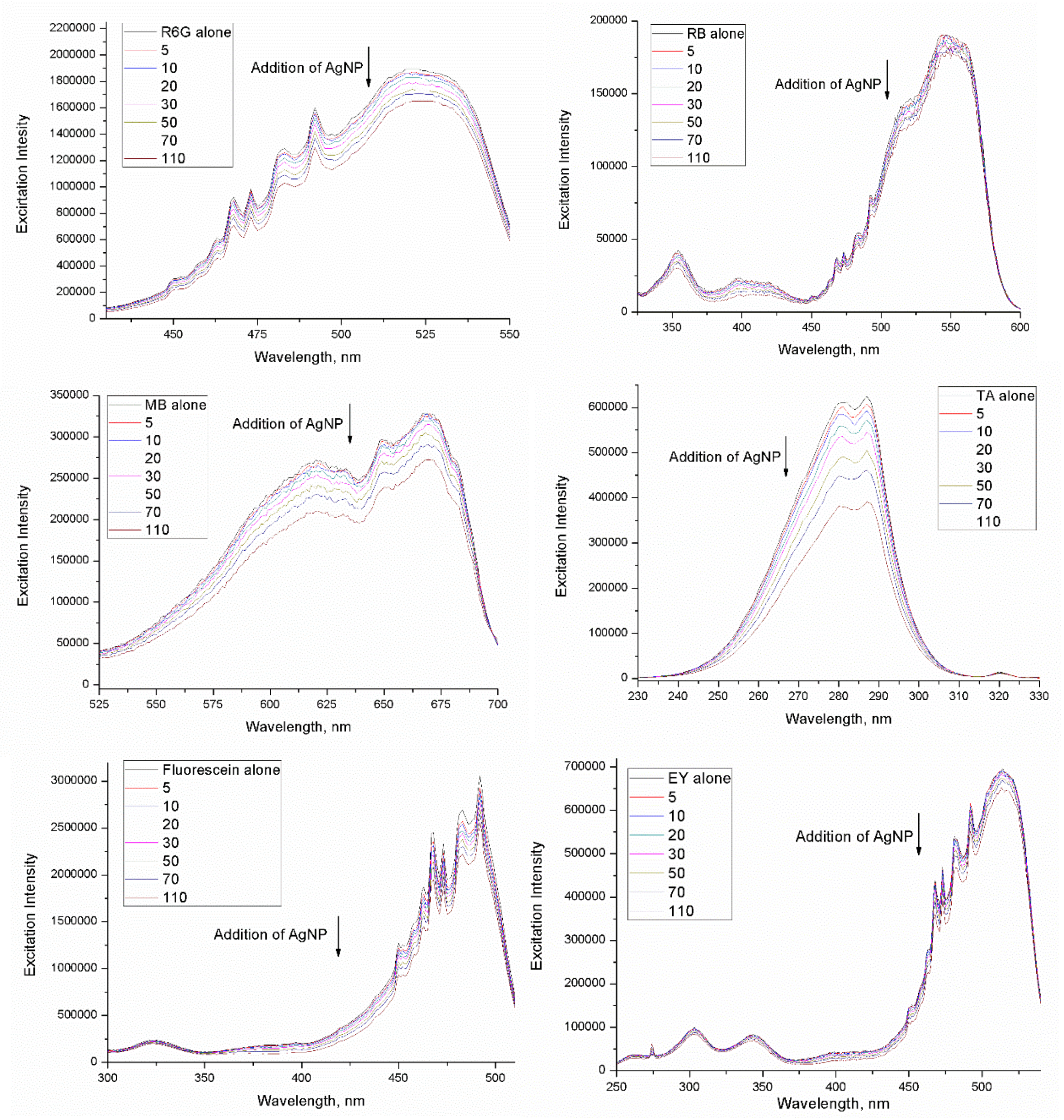

Figure S2. Excitation spectra of 1 μM dye solutions titrated with 240 pM AgNP. The numbers represent the volume (μL) of every single addition.

SI 3: Estimation of layer of dye molecules on AgNP surface

There are two methods to estimate the number of layers of dye molecules on AgNP surface. Using R6G as a representative, there is a big difference between the two methods. It is extensively discussed in our previous report [1].

First, agglomerates of roughly 310 nm were observed containing several "micelle" like structures. Each structure is around 170 nm in diameter, indicating that the radius of the micelle is 85 nm. The original AgNP's radius is 12 nm as unveiled by TEM. So, the actual thickness of the R6G molecules is 73 nm. Assuming that all R6G molecules align perpendicularly to the AgNP surface and a dynamic thickness/length of a single R6G is 2 nm, there will be about 36 layers of R6G molecules.

The second method assumes R6G as a cuboid (Figure S3). When they are assembled tightly, there is a distance of 0.3 nm between neighboring molecules, as indicated by the dashed lines. Therefore,

the dimension of an R6G molecule as "building unit" is estimated to be 2.0 nm × 1.4 nm × 0.7 nm for this ideal packing model in the micelles.

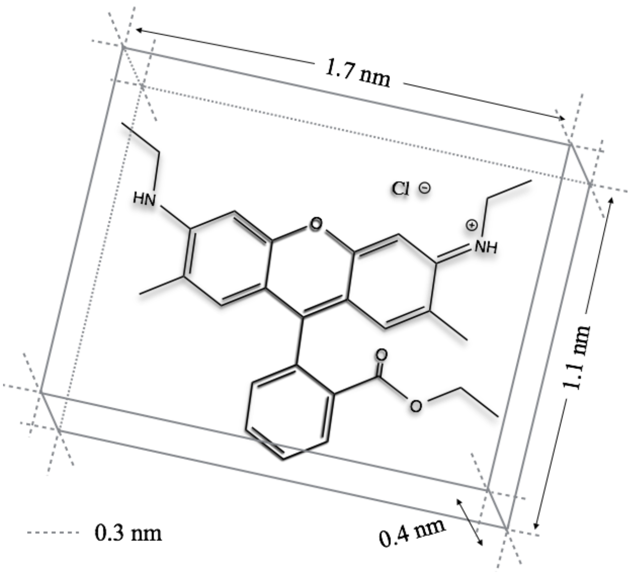

Figure S3. A cuboidal R6G molecule with a dimension of 1.7 nm × 1.1 nm × 0.4 nm. Extended dashes of 0.3 nm indicate the minimal distance between neighboring R6G molecules in the assembly. This yields an actual dimension of 2.0 nm × 1.4 nm × 0.7 nm within the assembly.

In the case that R6G molecules align perpendicularly to the AgNPs layer by layer on the surface of AgNP, the number of R6G molecules and the dynamic size of the structure are estimated in Table S1. The number of R6G molecules of any layer is estimated by dividing the surface area of that layer of the micelle (the initial "micelle" is the 24 nm AgNP) by the area of a R6G molecule (1.4 x 0.7 ≈ 1 nm^2).

Table S1. Theoretical estimation of the number of layers and total numbers of R6G molecules on AgNP surface in a micelle-like structure.

Layer	Size, nm	Surface area, $\times 10^3$ nm^2	Number of R6G in the layer, $\times 10^3$	Total number of R6G, $\times 10^4$
0	24	1.81	0.00	0.00
1	28	2.46	1.85	0.18
2	32	3.22	2.51	0.44
3	36	4.07	3.28	0.76
4	40	5.03	4.15	1.18
5	44	6.08	5.13	1.69
…	…	…	…	…
16	88	24.33	22.62	16.70
17	92	26.59	24.82	19.18
**18**	**96**	**28.95**	**27.13**	**21.90**
19	100	31.42	29.54	24.85
20	104	33.98	32.06	28.06
21	108	36.64	34.67	31.52
22	112	39.41	37.39	35.26
…	…	…	…	…
28	136	58.11	55.86	63.98
29	140	61.58	59.29	69.91
30	144	65.14	62.83	76.19

Note: Parameters: AgNP, d= 24 nm, surface area = $4\pi r^2$ = 1810 nm^2, hydrodynamic length for R6G, l = 2 nm. Area of R6G cross-section = width × depth = 1.4 nm × 0.7 nm = 0.98 $nm^2 \approx 1$ nm^2.

All R6G molecules whose fluorescence is quenched should be involved in the formation of the micelle-like structure with AgNP. Therefore, the number of R6G molecules quenched by a single AgNP is estimated by multiplying fluorescence quenching percentage with original ratio of R6G to AgNP. The results are listed in Table S2. In the case of 1 μM R6G, Stern Volmer type plot shows a saturation point of AgNP to R6G ratio around 6.78×10^5, where about 2.13×10^5 of R6G molecules (or approximate to 200,000 R6G molecules as used in the text) are quenched by every single AgNP. This matches 18 layers of R6G molecules.

Table S2. Number of R6G molecules whose fluorescence is quenched by a single AgNP, and their ratio, possible number of layers, as well as the dynamic diameter of the micelle-like structure.

[AgNP], $\times10^{-6}$ μM,	R6G/AgNP, $\times10^{6}$	AgNP/R6G, $\times10^{-6}$	F/F_0	% Quenching	Number of R6G per AgNP, $\times10^{5}$	Estimated number of layers
0.00	/	0.00	1.00	0.00	/	/
0.06	16.96	0.06	0.92	8.05	13.66	38
0.18	5.65	0.18	0.84	16.19	9.15	33
0.29	3.39	0.29	0.80	19.86	6.74	29
0.41	2.42	0.41	0.76	23.70	5.74	27
0.59	1.70	0.59	0.74	25.86	4.39	24
0.71	1.41	0.71	0.72	27.55	3.89	23
0.88	1.13	0.88	0.71	28.62	3.24	22
1.00	1.00	1.00	0.70	29.51	2.94	21
1.17	0.85	1.18	0.69	30.76	2.61	20
**1.46**	**0.68**	**1.47**	**0.69**	**31.42**	**2.13**	**18**
1.76	0.57	1.77	0.68	32.34	1.83	17
2.34	0.42	2.36	0.68	32.49	1.38	15
2.91	0.34	2.95	0.67	33.24	1.13	14
…	…	…	…	…	…	…
39.31	0.02	47.17	0.61	39.30	0.08	4
43.32	0.02	53.07	0.60	39.75	0.07	3
47.17	0.02	58.96	0.60	40.14	0.07	3
54.43	0.01	70.76	0.60	40.23	0.06	3
61.15	0.01	82.55	0.59	40.85	0.05	3

SI 4: Fluorescence lifetime measurement

Fluorescence lifetime of R6G in the absence or presence of different concentrations of AgNP is measured with the DS configurator that comes with the Horiba scientific instrument. The decay was analyzed using "Decay analysis software v 6.6". Nanoled-450 (wavelength of 451 nm) was used as excitation light and 7500 counts were collected. Nanopure water was used as a control. R6G has a fluorescence lifetime around 4.0 ns regardless of the presence of AgNP, indicating that the fluorescence quenching of 1 μM R6G by citrated-coated AgNP is static quenching, instead of collisional or dynamic quenching. As shown in Figure S7, two decay examples are shown.

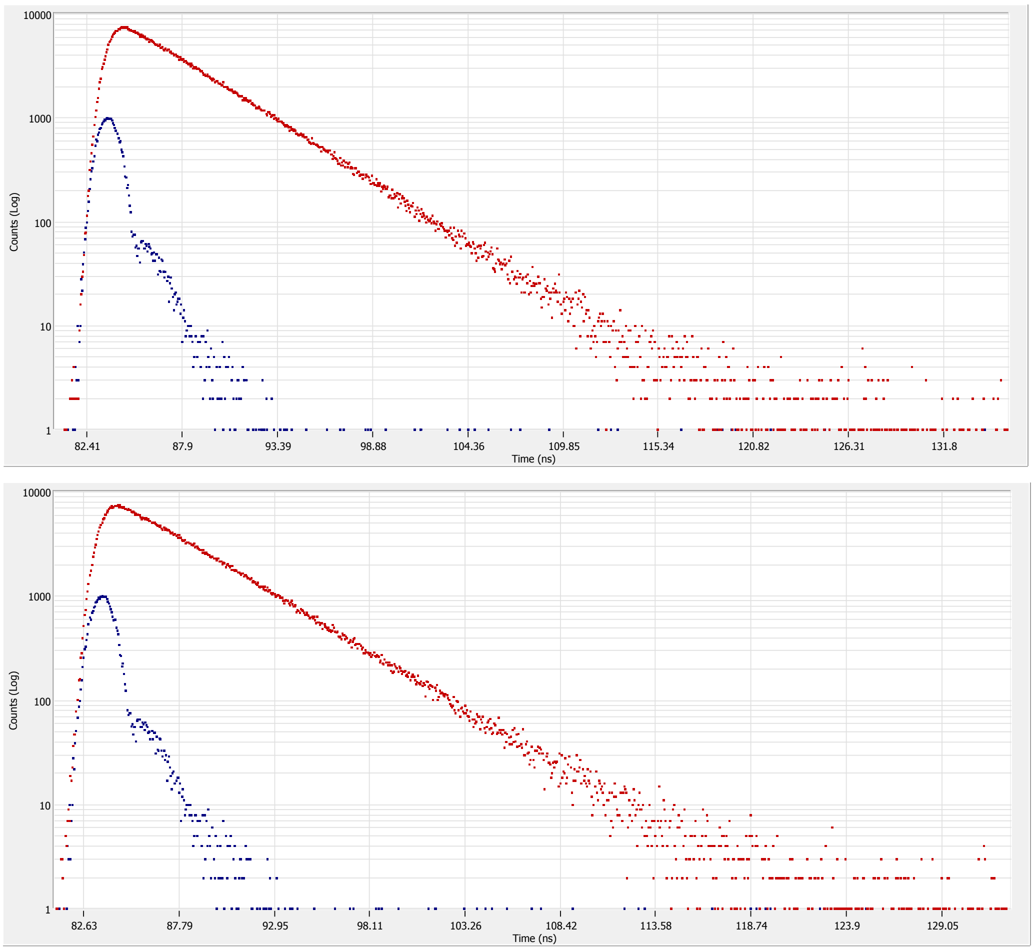

Figure S4. Fluorescence decay of 1 μM R6G alone (above) and that in the presence of citrate-coated AgNP (below, R6G to AgNP ratio of 8.48×10^5).

Reference

[1] H. Deng, H. Yu, Self-assembly of rhodamine 6G on silver nanoparticles, Chem. Phys. Lett. 692(16) (2018) 75-80.
